# The Role of Ozone as an Nrf2-Keap1-ARE Activator in the Anti-Microbial Activity and Immunity Modulation of Infected Wounds

**DOI:** 10.3390/antiox12111985

**Published:** 2023-11-08

**Authors:** Marianno Franzini, Luigi Valdenassi, Sergio Pandolfi, Umberto Tirelli, Giovanni Ricevuti, Salvatore Chirumbolo

**Affiliations:** 1 Italian Scientific Society of Oxygen-Ozone Therapy (SIOOT), 24020 Bergamo, Italy; marianno.franzini@gmail.com (M.F.); luigi.valdenassi@unipv.it (L.V.); sergiopandolfis2@gmail.com (S.P.); 2 Tirelli Medical Group, 33170 Pordenone, Italy; utirelli@tirellimedical.it; 3 Department of Drug Science, University of Pavia, 27100 Pavia, Italy; giovanni.ricevuti@unipv.it; 4 Department of Engineering for Innovation Medicine, University of Verona, 37125 Verona, Italy

**Keywords:** ozone, immunity, Nrf2, macrophage, macrophage training, tolerance

## Abstract

Ozone is an allotrope of oxygen, widely known to exert an anti-oxidant potential. The ability of low, controlled and standardized doses of ozone in the ozone adjunct treatment of bacterial infections, which occur in wounds, is engaging clinical research to deepen the role of ozone in eradicating even multidrug-resistant bacteria. Ozone activates the nuclear factor erythroid 2-related factor 2 (Nrf2), and this activation triggers a complex cascade of events, which ultimately leads to macrophage training and an improvement in their ability to operate a clearance of bacteria in the patient’s anatomical districts. In this review, we try to elucidate the recent evidence about the mechanisms with which ozone can actually remove bacteria and even multi-drug-resistant (MDR) bacteria, accounting on its complex ability in modulating immunity.

## 1. Introduction

Ozone is an allotrope of oxygen that has been extensively used in several medical settings as an adjunctive therapy, particularly in the treatment of painful chronic inflammations and degenerative musculoskeletal disorders [1]. In recent years, ozone (O_3_) has also been introduced in the medical complementary therapy of infected post-surgical or traumatic wounds, and has been suggested as a reliable tool to address the huge concern of multidrug-resistant (MDR) bacteria, though usually at high doses and in dermatological formulations for topical use [2,3,4]. The chemical ability of ozone to rapidly damage and kill any kind of microbial organism, encouraged the use of ozonated oil and ozonated water against bacteria, as they reported negligible side effects on treating the wounds of infected patients [5,6].

Ozone is widely known as an anti-oxidant molecule [7,8], and, as detailed below, its ability to activate the nuclear factor erythroid 2-related factor 2/Kelch-like ECH-associated protein 1/Antioxidant Responsive Element (Nrf2/Keap1/ARE) pathway [9,10] has a consequence on its potential in modulating the immune response of cells, including the anti-microbial activity. As a matter of fact, the activation of the nuclear factor erythroid 2-related factor 2 (Nrf2) can improve the phagocytic ability of macrophages through several mechanisms, including the simplest anti-oxidant response. Nrf2 is a transcription factor that plays a key role in regulating the expression of various antioxidant enzymes, such as haeme oxygenase-1 (HO-1) and NAD(P)H-quinone oxidoreductase 1 (NQO1) [11,12,13]. When Nrf2 is activated, it upregulates these enzymes alongside many other anti-oxidant factors, such as catalase, superoxide dismutase, and glutathione peroxidase, leading to an increased detoxification of reactive oxygen species (ROS) and other harmful molecules.

When this reduction in oxidative stress occurs within innate immune cells, such as macrophages, ozone can enhance, via secondary metabolites known as ozonides, their immune and anti-microbial function, including phagocytosis [14]. Moreover, the activation of the Nrf2/keap1/ARE pathway can also suppress the production of pro-inflammatory cytokines and chemokines, via interplay with the inflammasome NLRP3 [15].

By reducing inflammation, Nrf2 activation can contribute in the creation of a less hostile environment for macrophages, allowing them to perform their phagocytic functions more efficiently. Therefore, as a common activator of the Nrf2 expression and function, ozone can be suggested as a good approach in the clearance of systemic bacterial infections, including multi-drug-resistant (MDR) bacteria. This allows clinicians to consider ozone, being a possible trigger of the Nrf2 expression, a trustworthy activator of the anti-microbial response by innate immunity. Furthermore, Nrf2 activation can promote autophagy, a cellular process that helps innate immunity to eliminate damaged organelles and intracellular pathogens [16]. Autophagy is closely related to phagocytosis, as both processes involve the removal of cellular debris and pathogens. Nrf2-mediated autophagy can enhance the ability of macrophages to remove intracellular pathogens [17,18,19].

The whole endowment of these Nrf2-related processes enables ozone to behave as a strong antibacterial compound, even in a systemic context, such as the bloodstream, alongside its effectiveness in topical formulations. In addition, Nrf2 activation can protect macrophages from oxidative stress-induced cell death, thereby increasing the number of viable macrophages available for phagocytosis. This ensures a more robust phagocytic response [20].

Actually, the role of Nrf2 is of paramount importance to comprehend the ability of ozone to counteract a septic shock following an infected wound. Nrf2 activation can influence the expression of cell surface receptors involved in phagocytosis, such as scavenger receptors and complement receptors. The increased expression of these receptors can enhance the recognition and binding of pathogens by macrophages [21]. Finally, Nrf2 activation may shift macrophages towards the M2 phenotype, which is associated with anti-inflammatory and tissue repair functions. M2 macrophages are generally more efficient in the phagocytosis of certain targets, particularly those related to tissue repair and remodeling [22].

In summary, Nrf2 activation enhances the phagocytic capacity of macrophages by reducing oxidative stress, inflammation, and cell death, promoting autophagy, increasing the expression of phagocytic receptors, and potentially shifting macrophage polarization towards a phenotype, leading to phagocytosis. This makes Nrf2 a promising target for therapeutic interventions aimed at improving the immune response, especially in conditions where phagocytosis is crucial, such as infections and tissue repair.

The thorough anti-microbial activity of ozone closely depends on its widely known anti-oxidant hallmark.

This would mean, therefore, that the final cut of the anti-microbial effect held by ozone lies on its anti-oxidant property.

Even at its lowest doses, ozone is able to activate the signaling pathway leading to the expression of anti-oxidant and scavenging enzymes via the Nrf2/Keap1/ARE pathway [9]. Harvey and colleagues reported, some years ago, in a chronic obstructive pulmonary disease (COPD) model, that the role of Nrf2 is crucial for the improvement of bacterial clearance by macrophages [23]. The way by which the activated Nrf2-mediated signaling enhances macrophage phagocytosis of bacteria involves the Macrophage Receptor with Collagenous Structure (MARCO), which is even potentiated, in this role, by sulforaphane [23]. In the paper by Harvey et al., sulforaphane was used as an inducer of Nrf2 activation, a molecule which is present in broccoli to target and activate the Nrf2-mediated signaling [23]. The overexpression of MARCO enhances bacterial phagocytosis from macrophages [24], as MARCO is a class A scavenger receptor in macrophages that binds and uptakes only Gram-positive and Gram-negative bacteria, oxidized by low-density lipoproteins (LDL) and environmental particles, except for yeasts. Nrf2 directly regulates the expression of MARCO, therefore a close interplay between Nrf2 activation and MARCO expression does exist [23].

Interestingly, the scavenger receptor MARCO modulates also the activity of major antigen presenting (APC) cells, as much it modulates the TLR-induced response from dendritic cells [25]. This would mean that the activation of MARCO by Nrf2 allows the setting of a functional bridge between innate and adaptive immunity [25]. Once innate immunity shifts towards the activation of an antibody-mediated immune response and of adaptive cell immunity, the bacterial clearance is highly improved.

In this paper we will address the role of ozone and its major ozone-derived metabolites, known as ozonides [4,9], in counteracting the development of septic infections from post-surgical or traumatic wounds, trying to shed light on two different mechanisms with which ozone kills bacteria: (a) a chemical, direct effect of ozone; (b) an indirect effect mediated by low-dosed, ozone-derived mediators and targeting the Nrf2/Keap1/ARE system. In our opinion, and following some evidence published elsewhere, this ability may successfully address the huge concern of MDR bacteria.

## 2. Ozone’s Ability to Kill Microbes, Including MDR Bacteria: Role of the Nrf2-HO-1-CO Axis and M2-Macrophages

**Figure 1:**
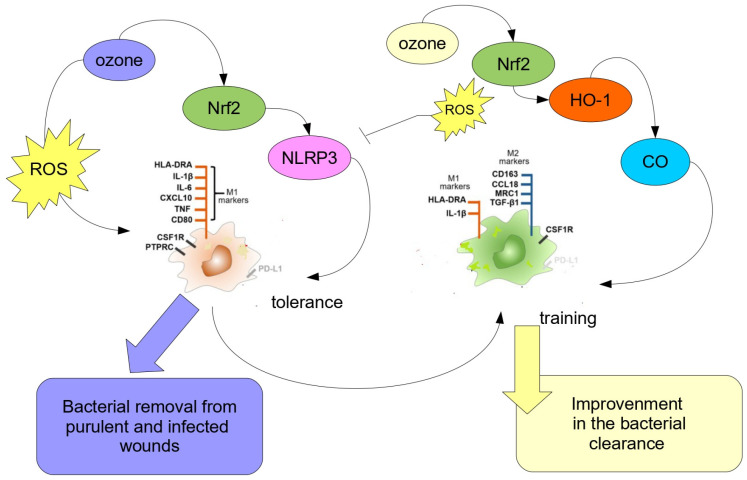
Cartoon showing the activity of ozone against bacterial infection in wounds. See text for details.

Figure 1 summarizes the different approaches to address bacterial infections in post-surgical or traumatic wounds, also involving MDR bacteria, which are highly sensitive to ozone, despite their MDR phenotype. The use of topical ozone (left) accounts on the toxic, pro-oxidant features of this oxygen allotrope, in which their activity relies on the pro-oxidant activity of reactive oxygen species (ROS) and the subsequent activation of the inflammasome NLRP3 and of M1 polarization (via NF-κB). Ozone acts as a disinfectant, killing bacteria colonizing the wound surface. Hormetic ozone (right, pale yellow) inhibits the inflammasome activation and induces, via Nrf2-HO-1-CO, the skewing of M1/M2, thus promoting macrophage training. Fundamentally, the concurrent actions of topical ozone and ozone injected via oxygen-ozone major autohaemotherapy (O_2_-O_3_-MAHT) should be used together in order to prevent bacterial-induced sepsis, particularly in those circumstances where an MDR bacterial infection is present.

The description reported in the previous chapter would suggest that the ability of ozone to efficiently remove bacteria depends on the complex role exerted by the Nrf2/Keap1/ARE, i.e., by the anti-oxidant property of ozone. It is possible that during the administration of modest doses of ozone via major autohemotherapy (or auto-hemoinfusion) (O_2_-O_3_-MAHT), red blood cells undergo a moderate hemolysis, releasing heme groups, which triggers the activation of HO-1 [26,27].

About 2% of the ozone injected into the plasma with the O_2_-O_3_-MAHT is converted into the electrophilic alkenal 4-trans-hydroxynonenal (4-HNE), via the lipoperoxidation of ω6-polyunsaturated fatty acids (PUFAs) [28], following Henty’s law and considering that a dose close to 45 μg/mL O_3_ in the gaseous mixture with oxygen at 25 °C, should correspond to about 8 μg/mL O_3_ in the blood at 37 °C; a classical O_2_-O_3_-MAHT might elicit about 3.0 μM 4-HNE, which, at this concentration, exhibits an anti-inflammatory activity [29]. This dose of 4-HNE is able to activate the Nrf2-mediated immune regulation, alongside inhibiting the activation of the inflammasome NLRP3 [29,30].

Low doses of ozone in the medical adjunct treatment, by inducing a mild activation of 4-HNE, should trigger the expression of Nrf2 via 4-HNE, therefore causing the expression of thioredoxin reductase 1 and protecting cells from the subsequent apoptotic signal promoted by H_2_O_2_ [30]. The same 4-HNE at 3.0 μM inhibits innate immune cell pyroptosis [29].

Moreover, the activation of Nrf2 induces the synthesis of HO-1 [31], which catalyzes the degradation of heme groups into biliverdin (then reduced to bilirubin) and carbon monoxide (CO), a chemical mediator that activates a guanylate cyclase in endothelia and exerts effects comparable to nitric oxide (NO), regulating vascular physiology, neuronal signaling and the modulation of apoptosis [32]. The gaseous mediator CO is able to inhibit the activation of NF-κB, which is in a complex equilibrium with the activity of Nrf2, inasmuch CO suppresses the bacterial lipopolysaccharide (LPS)-induced phosphorylation and degradation of IκBα, therefore inhibiting NF-κB signal transduction [33,34].

This anti-inflammatory activity, induced by the cascade O_3_-Nrf2-HO-1-CO, leads to the formation of the CO-mediator and the blocking of a pro-inflammatory signal via the activation of the NF-κB-mediated signaling. Carbon monoxide is a powerful signal able to educate macrophages in finely sensing bacteria, as microbial clearance to prevent septic shock is a crucial process in the cell response to stress and damage [35]. The gaseous transmitter CO promotes the production and release of ATP from colonizing bacteria, enabling them to activate the macrophages’ inflammasome NALP3 and allowing bacterial killing [35]. At the same time, CO promotes M1/M2 skewing via HO-1, enhancing macrophage polarization towards the M2 phenotype [36]. Interestingly, at least in rats, a subcutaneous injection of ozone induces the M2-phenotype polarization of macrophages [37].

The classical activation of naïve macrophages into the M1-pro-inflammatory phenotype occurs via Th1-related stimuli, such as LPS, IFN-γ and TNF-α, using glycolysis-driven metabolic pathways, whereas the alternative activation, leading to the M2 polarization, occurs with cytokines such as IL-4, IL-13, IL-10 and TGF-β, which are downstream signals of oxidative phosphorylation and lipoperoxide production pathways [38]. During bacterial-driven inflammation, the skewing towards the M2-phenotype is mandatory to ensure the removal of apoptotic cells, cell debris and pathogens, as M2-macrophages have an enhanced ability in phagocyting bacteria with respect to M1, and even MDR bacteria such as MRSA [39,40]. Sepsis is prevented by proper interplay between M1 and M2 phenotypes [41].

The removal of apoptotic cells (efferocytosis) is fundamental to ensure the correct process of bacterial eradication via the engulfment of apoptotic infected cells. Despite microorganisms having different strategies to escape from efferocytosis by interfering with apoptosis and/or macrophage skewing, some substances, such as taurine chloramine, are considered able to improve bacterial clearance [42,43,44].

Moreover, to remove bacteria from a wound using ozone, a wearable system, made by a dressing consisting of a multi-layered system, enabled being able to widely display the ozone produced by a portable and reusable ozone-generator via a tube, which was also reported in [44]. However, this strategy is uncommon, less feasible and uncomfortable, and we only reported it here for completeness.

This leads us to question if the enhancement in phagocyting bacteria by M2-macrophages is sufficient to eradicate bacteria and also MDR bacteria. Probably not, but the increase in efferocytosis, upon an extensive ozone treatment, should reach a promising result [45,46,47].

The main process that an oxidant-inducing stress such as O_3_ can start, via the Nrf2-HO-1-CO pathway, is training immune cells in improving their anti-bacterial ability, subsequently ameliorating the bacterial clearance in the infected wounds.

How ozone is able to “educate” the innate immunity in enhancing the anti-microbial function is not directly linked with the action of ozone in the organism, but with the ability of its major mediators, alkenals and lipoperoxides (LPOs), such as 4-HNE and malonyl dialdehyde (MDA), to modulate important signaling pathways related with Nrf2 activation.

**Figure 2:**
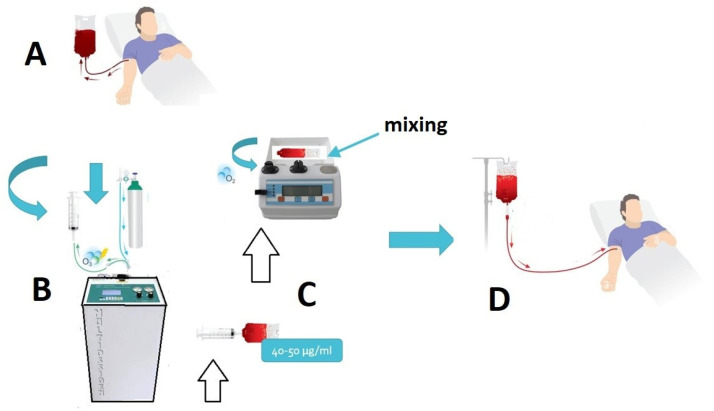
The main steps in the O_2_-O_3_-MAHT according to the SIOOT protocol. (**A**) A volume of 200 mL of peripheral blood is withdrawn and put in a sterile SANO3 disposable, certified bag; (**B**) ozone is prepared via a Multiossigen Medical 98 HCPS device (Gorle, Italy); its concentration measured in an O_2_/O_3_ mixture with an endowed spectrophotometer at 253.7 nm, reaching a final concentration of 40–50 μg/mL, depending on the clinical use; (**C**) ozone is collected in a 50 mL sterile, disposable syringe directly from the device and injected into the blood-collection bag, then gently mixed for 2 min; (**D**) the ozonated autologous blood is transferred to the patient.

Figure 2 shows the major steps in the O_2_-O_3_-MAHT.

## 3. Ozone as a Trainer of the Anti-Bacterial Learning of Immune Cells?

The role of oxidative stress has paramount importance in leading the cell to a certain outcome upon any bacterial invasion. An example of this is the activity of the antibiotic fluoroquinolone, which binds to the bacterial targets’ DNA gyrase and topoisomerase IV. In this circumstance, oxidative stress, produced by macrophages, in particular, M1 phenotypes, downregulates these targets and therefore drastically reduces the activity of the antibiotic against bacteria, and therefore contributes in the creation of resistant strains [48].

The challenging struggle against bacteria held by innate immune cells, particularly by macrophages, when caused by a high exposure to LPS, induces the emerging of tolerant phenotypes [49]. Tolerant macrophages exhibit a lower phagocytic activity in response to receptors such as Fc, C3b and mannose receptors, yet the LPS-tolerant macrophages have an increased expression of antibody receptors and production of H_2_O_2_, indicating an improvement in bacterial fighting [50].

The excessive production of ROS, RNS and peroxynitrites, exacerbates the antibiotic tolerance, as reported for methicillin-resistant *Staphylococcus aureus* (MRSA), and worsens the macrophage tolerance, making these cells perfect hosts of living bacteria [51]. This should suggest that a huge oxidative stress response, as occurring in inflammation and in the M1-induced defense, if not rapidly quenched by the skewing towards M2 phenotypes, may generate a burdensome bacterial antibiotic tolerance [51].

In this sense, it is particularly intriguing how the anti-inflammatory activity of ozone works, which can be induced by a systemic injection of ozone via O_2_-O_3_-MAHT [45]. Ozone helps immunity in achieving the correct equilibria between M1 and M2 activity, thereby preventing sepsis. The anti-inflammatory ability elicited by ozone and its lipid peroxides (ozonides), directly coming from its anti-oxidant potential, has an educational (training) role towards the innate immunity, allowing the creation of much more skilled macrophages against bacteria [52].

Macrophage training typically refers to the process of educating or modulating macrophages, which are a type of white blood cell in the immune system, to enhance their function or redirect their activity in a desired way. Macrophages play a crucial role in the immune response by engulfing and digesting foreign invaders, dead cells, and cellular debris. They also contribute to tissue repair and the regulation of immune responses. The simplest way to “educate” macrophages is polarization, as these cells can be polarized into different functional states based on their microenvironment.

While tolerance and trained immunity are two different sides of the same coin, low doses of a stressor, for example, LPS, in a persistent activation, induces, on the contrary, a priming state, which enables innate cells to respond better to the next bacterial insult, whereas, as previously indicated, a repeated or prolonged exposure to high doses of LPS induces a tolerant state, inducing a macrophage reprogramming [53,54].

Oxidized lipoproteins (oxLDL), which are induced by ozone during an ozone adjunct treatment, can trigger macrophage training. When macrophages are trained by oxLDL, they switch from a glycolytic pathway to a mTOR-dependent oxidative phosphorylation with the induction of ROS, which represent fundamental signaling molecules for macrophage training [55].

The two main polarization states are M1 and M2. M1 macrophages are pro-inflammatory and involved in fighting infections, while M2 macrophages are anti-inflammatory cells and play a role in tissue repair and immune regulation. Researchers can manipulate macrophage polarization through the use of cytokines, growth factors, or other signaling molecules. Training macrophages to become more efficient at phagocytosis (the process of engulfing and digesting foreign particles) can be useful in fighting infection. This can be achieved through exposure to various substances that boost their phagocytic activity.

Research in macrophage training is a growing field with potential applications in immunotherapy, vaccine development, and the treatment of various diseases, including cancer and autoimmune disorders. The specific techniques and methods used can vary depending on the intended outcome and the underlying medical condition being addressed. Ozone can induce macrophage training, with an increasing macrophage recruitment if used in low doses and subjected to repeated exposure [56].

## 4. The Challenging Role of Ozone in Fighting against MDR Bacteria

**Figure 3:**
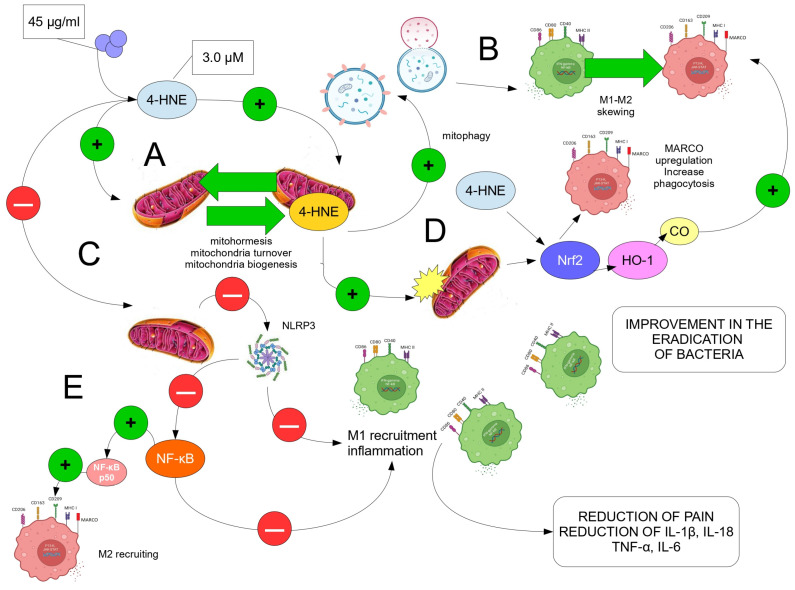
The hypothetical mechanisms by which ozone, injected via a systemic approach (bloodstream, with O_2_-O_3_-MAHT), is able to drive immunity towards a possible complete clearance of bacteria in infected wounds, giving encouraging suggestions also for MDR bacteria and setting an anti-inflammatory and skilled M2-immune response. (**A**) A dose of 45 μg/mL O_3_ in O_2_/O_3_ gas mixture, once injected into the blood, gives rise to about 3 μM 4-HNE, from ω6-PUFA peroxidation, a dose able to exert an anti-inflammatory action [29]. This dose promotes mitohormesis mitochondria turnover and biogenesis, via the contribution of also mitochondria-produced 4-HNE, and, moreover (**B**) it promotes the optimal mitochondria autophagy, which supports the M1-M2 skewing and the related immune regulation. (**C**) The alkenal 4-HNE inhibits, at 3 μM, the activation of the inflammasome NLRP3, therefore downregulating M1-driven inflammation and the subsequent maturation of the cytokines IL-1β and IL-18, alongside other pro-inflammatory cytokines. (**D**) The 4-HNE-mediated activation of Nrf2 triggers the HO-1/CO-mediated M1/M2 skewing and the Nrf2-caused upregulation on the M2 marker MARCO, which enhances the ability of these cells to phagocytize bacteria. (**E**) Finally, the NLRP3 inhibition blocks the activation of NF-κB, allowing only the activity of NF-κB p50, involving the recruitment of M2 macrophages and their immune training. Green circles: activation or promotion; Red circles: inhibition.

Figure 3 summarizes the role of ozone, used in a systemic way, e.g., via blood, in fighting against infectious bacteria, including MDR bacteria.

At this point of the debate, we are able to assess that the ability of ozone to reach a complete bacterial clearance, even if microorganisms are MDR bacteria, may depend on the particular methodological protocol used to eradicate microbes from the infected wounds.

As an example, the protocols recommended by the Italian Scientific Society of Oxygen–Ozone Therapy (SIOOT) to perform the adjunct medical treatment with ozone against bacteria in infected wounds, include both the use of topical ozone (either 5–10% ozonated water or ozonated oil or other olefinic formulation), and the introduction of the major autologous hemoinfusion (O_2_-O_3_-MAHT), where ozone is injected in the autologous peripheral blood via a sterile, disposable transfusion bag (Figure 2). Topical ozone can destroy bacteria directly, whereas ozone in the blood, which is rapidly quenched (within few minutes) via the plasma anti-oxidant system, will elicit the generation of ozonides able to modulate the immune response [4,57]. The ability of ozone to completely eradicate infectious bacteria from wounds, also including MDR bacterial infection, should account on both the chemical (pro-oxidant) activity of topical ozone on wounds and the hormetic action of systemic ozone on the complex signaling machinery of the immune response to stress, as summarized in Figure 3.

Recent evidence reported that ozone is able to kill methicillin-resistant strains of *Staphylococcus aureus* (MRSA), as ozonated oil can completely block *S. aureus* growth within 5 min and ozonated water within 1 min [5,6]. Ozone actually destroys *S. aureus* and *Bacillus cereus* bacteria in ozone-treated water, as reported by evaluating the change in the oxidation reduction potential (ORP). Ozonated water with ORP higher than 500 mV showed the ability to completely remove *S. aureus* and *B. cereus* biofilms within 24 h [58]. Furthermore, ozone showed a comparable potential with chlorhexidine in reducing *Pseudomonas aeruginosa* growth, as ozonated water caused a 7.42 log reduction of *P. aeruginosa* biofilms, similar to the chemical disinfectant [59]. Although ozone shows great ability in killing bacteria on infected skin and wounds, the majority of reports showing the efficacy of ozone against microbes regards gas ozone in an indoor environment.

A recent paper by Epelle et al. [60] wondered if gas ozone is more efficacious than ozone dissolved in water (ozonated water) to eradicate bacteria under similar conditions, i.e., ozone concentration, duration of ozone exposure, and temperature. By testing ozone on major bacterial strains, i.e., *Escherichia coli* NTCC 1290, *Pseudomonas aeruginosa* NCTC 10332, *Staphylococcus aureus* ATCC 25923 and *Streptococcus mutans*, the authors showed that gaseous ozone exhibited a better performance among all the investigated microorganisms, except for *S. aureus*, for which gas (10 ppm, approximately 10 μg/mL) into water works better in killing these Gram-positive bacteria [60]. Yet, this evidence supports the role of ozone as a noxious chemical component against bacteria, therefore representing a possible option for infected patients.

It is particularly clear that ozone can cause the lysis of the bacterial membrane, and that relatively low doses of ozone, though not always being able to inhibit bacterial growth, interferes with the bacterial cell survival, as reported for *S. aureus* ATCC 6538, *P. aeruginosa* ATCC 15442, *Escherichia coli* ATCC 25922 and *Acinetobacter baumannii* [61].

The recent contribution by Sottani et al. addressed the role of ozone as an indoor disinfectant in hospitals and healthcare structures, reporting that 6 ppm O_3_ for at least 1 h, aside from UV application, could drastically reduce the rate of infection of *Clostridium difficile* and many MDR bacteria [62].

Bong et al. demonstrated that 500 ppm ozone for 15 min were able to remove completely carbapenem-resistant *Klebsiella pneumoniae*, carbapenem-resistant *Pseudomonas aeruginosa*, and carbapenem-resistant *Acinetobacter baumannii* [63]. The majority of these results, despite regarding the use of indoor gas ozone rather than topical ozone applied to trauma or post-surgical infected wounds, represent possible, encouraging evidence to use ozone as a disinfectant, even in suitable and available formulations. However, the efficacy of ozone, as a potent pro-oxidant molecule, remains.

Low doses of ozone cannot be included in the consideration that the effect of ozone, in this circumstance, can be explained only accounting on its strong pro-oxidant effect when injected into a patient. Despite ozone inducing the production of potentially toxic lipoperoxides (LPOs), such as alkenals from PUFAs (4-HNE and 4-HHE), malonyl dialdehyde (MDA) and several oxysterols from cholesterol, by activating the generation of reactive oxygen species (ROS), such as hydroxyl radicals (OH•) from water, the levels of these metabolites are quite negligible, as ozone disappears within 4 min at 37 °C and at 45 μg/mL O_3_ (following the SIOOT protocol for O_2_-O_3_-MAHT) [57]; the concentration of 4-HNE, according to Henry’s law, is as high as 3.0 μM, which is a dose exerting an anti-inflammatory and anti-oxidant effect [29].

Low doses of ozone activate Nrf2 in a way that triggers neither a pro-inflammatory action by the NLRP3 inflammasome [29], nor an activation of NF-κB [33].

This circumstance promotes M1/M2 skewing, as previously described in this review, which in turn elicits an “educational training” of the immune cells towards an improvement in killing bacteria.

M2 macrophages are generally associated with tissue healing, immunoregulation, and resolution of inflammation, rather than direct bacterial killing. However, M2 macrophages are not specialized for bacterial killing. They may still perform phagocytosis, including the uptake of dead bacteria or cellular debris. This process helps in maintaining tissue homeostasis and clearing away remnants of bacterial infections after M1 macrophages have dealt with the active and painful infection. Furthermore, M2 macrophages are often found in damaged or inflamed tissues, where they contribute to tissue repair and remodeling. They can help clear away debris and support the reconstruction of damaged tissue. This is in contrast to M1 macrophages, which are more focused on eliminating pathogens like bacteria, at least in a first stage. Finally, M2 macrophages can also modulate the immune response by secreting anti-inflammatory cytokines and promoting the development of regulatory T cells (Tregs). This regulatory function helps prevent excessive immune reactions that could lead to tissue damage.

In summary, M2 macrophages are not primarily involved in the direct killing of bacteria. Instead, they play essential roles in regulating the immune response, promoting tissue repair, and resolving inflammation, driving the immune response to a complete clearance of bacteria. The killing of bacteria is typically carried out by M1 macrophages, which are pro-inflammatory and have more potent bactericidal capabilities.

Driving inflammation towards its resolution means, fundamentally, to remove any damage and pathogen-associated molecular patterns from the immune microenvironment, enhance phagocytosis to rapidly reach the bacterial clearance, ask for the active participation of adaptive immunity and repair damaged cells and tissue, promote survival functions, and put induce the finalization of autophagy and apoptotic signals.

Therefore, the anti-inflammatory effect held by ozone has not to be interpreted as a blockage of the inflammatory response towards bacteria, but as a refinement, an improvement, and a more keen and focused activity of the immune response towards the challenging task to remove any noxious bacterium from the inflammation milieu.

This task is particularly attributed to a kind of ozone acting in a systemic way, therefore to a “hormetic” ozone.

Hormesis is a biological phenomenon in which exposure to low doses of a stressor or toxin can actually be beneficial or stimulate adaptive responses in an organism, leading to improved resilience and health. This concept has been observed in various biological systems, including plants, animals, and humans. Ozone exposure can sometimes demonstrate hormetic effects, depending on the dose and context of exposure.

Joining topical ozone on wounds with ozone via autohemotherapy (SIOOT protocol), should be considered a thorough approach to eradicate bacterial infections and preventing sepsis in ulcerous and infected post-operative wounds, even with MDR bacteria.

## 5. Conclusions

Ozone, at low, standardized doses, showed the ability to cleanse post-surgical and traumatic-infected wounds from sepsis, and led to the complete clearance of infectious bacteria, including MDR bacteria, via the concurrent use of ozone dermatological formulations and O_2_-O_3_-MAHT. The ability of ozone in removing bacteria from wounds would suggest its use in the problem of antibiotic resistance. The possibility to overcome the huge concern of antibiotic resistance, particularly in the post-surgical setting, is a major advantage held by ozone as an adjunct medical treatment, and future consideration of this opportunity may significantly support medical healthcare in addressing nosocomial deaths and illness exacerbations due to bacterial infections, showing a concerning refractoriness to be completely eradicated. Antibiotic resistance is a growing global health concern that occurs when bacteria, fungi, or other microorganisms evolve to become resistant to the drugs (antibiotics) that are designed to kill or inhibit their growth. This resistance makes it more challenging to treat infections, as the antibiotics become less effective or completely ineffective. It is essential for both healthcare professionals and the general public to be aware of the consequences of antibiotic resistance and to take steps to use antibiotics responsibly. Therefore, ozone could be a remarkable opportunity to see this to fruition.

## Data Availability

No data to be reported.
